# Special Issue “Current Research for Heart Disease Biology and Therapeutics: 2nd Edition”

**DOI:** 10.3390/ijms26178709

**Published:** 2025-09-06

**Authors:** Claudia Kusmic

**Affiliations:** Institute of Clinical Physiology, National Research Council (CNR), 56124 Pisa, Italy; claudia.kusmic@cnr.it; Tel.: +39-050-315-2669

Cardiovascular disease (CVD) remains one of humanity’s most significant health challenges, causing more deaths each year than any other condition. In 2021, CVD accounted for more than 20 million deaths worldwide [1]—about one-third of all fatalities. This burden is projected to rise to over 35 million annually by 2050. Behind these numbers are aging populations, persistent risk factors such as hypertension, diabetes, and obesity, and deep disparities in healthcare access across regions [1,2,3]. These alarming statistics remind us that while the past decades have brought progress in prevention and treatment, this progress has not been enough.

Addressing this challenge requires not only determination but also imagination—an ability to rethink disease mechanisms, develop new therapies, and expand our vision of what is achievable in cardiovascular medicine. With this mindset, we present the second edition of *Current Research in Heart Disease Biology and Therapeutics*, which features a collection of eight articles. This edition highlights innovative studies and critical reviews that encompass advances in molecular discoveries, regenerative strategies, clinical decision-making, and environmental factors affecting cardiovascular health.

We extend our sincere gratitude to the authors, peer reviewers, and editorial team whose contributions have made this Special Issue possible. Together, their work reflects both the depth of scientific inquiry and the collaborative effort required to address one of the most complex health challenges of our time.

The contributions can be grouped into three major themes: molecular/epigenetic regulation, regeneration and repair, and clinical/environmental perspectives.

At the core of cardiovascular disease lies a complex network of molecular events that drive pathological remodeling and dysfunction. Two reviews in this issue explore how epigenetic regulation—including DNA methylation, histone modifications, and noncoding RNAs—along with fibroblast heterogeneity, reshapes the heart under stress [4,5]. The reviews highlight the impact of these processes on cardiac hypertrophy, fibrosis, and progression to heart failure, while also exploring the potential of epigenetic therapies to reverse maladaptive remodeling. Importantly, they address challenges such as specificity, off-target effects, and the transition of these therapies from research to clinical practice, highlighting the significant gap that needs to be bridged to move from the laboratory to patient care.

Equally compelling is new research on proteostasis and network biology. One communication investigates the interactome of the chaperone protein BAG3, which is a multifunctional regulator of protein quality control and mitochondrial integrity [6]. Mapping its connections in human cardiomyocytes reveals how dysruption in BAG3 may contribute to both ischemic and idiopathic cardiomyopathies. These molecular insights not only describe disease mechanisms but also highlight therapeutic targets where interventions could stabilize cardiac function or slow disease progression.

A second theme focuses on restoring the heart after damage occurs. Two reviews in this topic examine the regenerative potential of the myocardium, ranging from neonatal models with strong repair capacity to emerging strategies aimed at coaxing regeneration in the adult heart. Endogenous mechanisms, such as cardiomyocyte proliferation, progenitor cell activity, and neovascularization, are discussed in the context of therapeutic interventions designed to enhance these processes following myocardial infarction [7]. Complementing this, extracellular vesicles (EVs) derived from stem and progenitor cells are highlighted as a promising “cell-free” therapy. These vesicles can deliver bioactive cargo—ranging from RNAs to proteins—which present opportunities for modulating inflammation, fibrosis, angiogenesis, and survival after injury [8]. Engineering approaches, including hydrogel embedding and targeted surface modification, demonstrate how to overcome translational challenges. Together, these studies point toward a future where therapies go beyond halting progression and instead aim to rebuild functional myocardium.

While molecular and regenerative therapies hold the potential for transformative change, advancements in clinical practice are equally important. A systematic review featured in this issue challenges long-standing beliefs regarding heart transplantation in patients with muscular dystrophy. It shows that when selection is managed carefully, survival outcomes for these patients can be comparable to those of recipients without muscular dystrophy [9]. These findings expand treatment possibilities for groups that were previously considered untreatable, reminding us that innovation involves re-evaluating established clinical practices.

Other contributions look outward, expanding cardiovascular research into systemic and environmental domains. One review examines the vascular complications of COVID-19, emphasizing how endothelial dysfunction and pulmonary vasculopathy may put survivors at an increased risk of pulmonary hypertension and chronic thromboembolic disease [10]. Another study explores the impact of microgravity and space radiation on cardiovascular health, a consideration particularly relevant as human exploration extends beyond Earth [11]. These perspectives underscore the importance to understand and address cardiovascular biology in dynamic contexts.

The articles in this Special Issue highlight the richness and diversity of current research in heart disease biology and treatment. They remind us that progress stems from the convergence of various disciplines—molecular biology, regenerative medicine, clinical insight, and systemic understanding of the body.

Looking forward, several promising frontiers emerge. First, the integration of single-cell omics and advanced imaging appears poised to unravel the cellular heterogeneity and dynamic regulation that underlie cardiac pathology. Second, regenerative and cell-free therapies, though still in their early stages, hold the potential to transform patient outcomes by restoring lost function rather than merely slowing the progression of disease. Lastly, as global health challenges evolve—whether due to pandemics, demographic shifts, or even the demands of space travel—cardiovascular research must be prepared to anticipate and adapt to these new stressors.

We hope that the discoveries and insights presented here will inspire further innovation, foster collaboration across disciplines, and ultimately contribute to the development of more effective and equitable treatments. The second edition of this Special Issue serves not only as a collection of knowledge but also as an invitation to think boldly, challenge assumptions, and continue striving toward a future where the burden of cardiovascular disease is greatly diminished.

## References

[1] Di Cesare M., Perel P., Taylor S., Kabudula C., Bixby H., Gaziano T.A., McGhie D.V., Mwangi J., Pervan B., Narula J. (2024). The Heart of the World. Glob. Heart.

[2] Bozkurt B., Ahmad T., Alexander K.M., Bosak K., Breathett K., Fonarow G.C., Heidenreich P., Ho J.E., Hsich E., Ibrahim N.E. (2023). Heart failure epidemiology and outcomes statistics: A report of the Heart Failure Society of America. J. Card. Fail..

[3] Chong B., Jayabaskaran J., Jauhari S.M., Chan S.P., Goh R., Kueh M.T.W., Li H., Chin Y.H., Kong G., Anand V.V. (2024). Global burden of cardiovascular diseases: Projections from 2025 to 2050. Eur. J. Prev. Cardiol..

[4] Ho J.S.Y., Jou E., Khong P.-L., Foo R.S.Y., Sia C.-H. (2024). Epigenetics in Heart Failure. Int. J. Mol. Sci..

[5] Aguado-Alvaro L.P., Garitano N., Pelacho B. (2024). Fibroblast Diversity and Epigenetic Regulation in Cardiac Fibrosis. Int. J. Mol. Sci..

[6] Qu H.-Q., Wang J.-F., Rosa-Campos A., Hakonarson H., Feldman A.M. (2024). The Role of BAG3 Protein Interactions in Cardiomyopathies. Int. J. Mol. Sci..

[7] Fiorino E., Rossin D., Vanni R., Aubry M., Giachino C., Rastaldo R. (2024). Recent Insights into Endogenous Mammalian Cardiac Regeneration Post-Myocardial Infarction. Int. J. Mol. Sci..

[8] Guerricchio L., Barile L., Bollini S. (2024). Evolving Strategies for Extracellular Vesicles as Future Cardiac Therapeutics: From Macro- to Nano-Applications. Int. J. Mol. Sci..

[9] Politano L. (2024). Is Cardiac Transplantation Still a Contraindication in Patients with Muscular Dystrophy-Related End-Stage Dilated Cardiomyopathy? A Systematic Review. Int. J. Mol. Sci..

[10] Riou M., Coste F., Meyer A., Enache I., Talha S., Charloux A., Reboul C., Geny B. (2024). Mechanisms of Pulmonary Vasculopathy in Acute and Long-Term COVID-19: A Review. Int. J. Mol. Sci..

[11] Mircea A.A., Pistritu D.V., Fortner A., Tanca A., Liehn E.A., Bucur O. (2024). Space Travel: The Radiation and Microgravity Effects on the Cardiovascular System. Int. J. Mol. Sci..

